# Frequent attenders at risk of disability pension: a longitudinal study combining routine and register data

**DOI:** 10.1177/1403494819838663

**Published:** 2019-04-11

**Authors:** Tiia T.M. Reho, Salla A. Atkins, Nina Talola, Markku P.T. Sumanen, Mervi Viljamaa, Jukka Uitti

**Affiliations:** 1 Tampere University, Faculty of Medicine and Health Technology, Tampere, Finland; 2 Pihlajalinna Työterveys, Tampere, Finland; 3 Tampere University, New Social Research and Faculty of Social Sciences, Tampere, Finland; 4 Karolinska Institutet, Department of Public Health Sciences, Stockholm, Sweden; 5 Finnish Institute of Occupational Health, Tampere, Finland; 6 Clinic of Occupational Medicine, Tampere University Hospital, Tampere, Finland

**Keywords:** Primary healthcare, patient acceptance of healthcare, occupational health services, rehabilitation, disability evaluation

## Abstract

*Aims:* Frequent attendance in healthcare services is associated with ill-health and chronic illnesses. More information is needed about the phenomenon’s connection with disability pensions (DPs). *Methods*: The study group comprised 59,676 patients divided into occasional- (1yFAs) and persistent frequent attenders (pFAs) and non-frequent attenders (non-FAs). Odds ratios for DP were analysed for these groups taking into account preceding sickness absence days. The awarded DPs were obtained from the Finnish Centre for Pensions and data on primary care visits were obtained from Pihlajalinna, a nationwide occupational healthcare provider. *Results*: 1yFAs and pFAs have more DPs than non-FAs. During follow-up, 14.9% of pFAs, 9.6% of 1yFAs and 1.6% of non-FAs had a DP decision of any kind. pFAs receive more partial and fixed-term decisions than the other groups and most permanent DPs are granted to 1yFAs. Musculoskeletal disorders are the most common reason for illness-based retirement in all groups but 1yFAs and pFAs have proportionally more mental disorders leading to DP. The group of non-FAs, on the other hand, has more DPs granted based on neoplasms. Both 1yFAs and pFAs have an increased risk of DP but the effect is diluted after taking into account preceding sick-leave. ***Conclusions*: Frequent attendance of healthcare services, both occasional and persistent, is associated with increased risk of future DP. The association is linked to increased sickness absences. Frequent attenders should be identified and their rehabilitative needs evaluated. Frequency of consultation** **could be used in selecting candidates for early rehabilitation before sickness absences develop.**

## Introduction

Illness-based retirement represents a personal loss and a social and economic challenge. In 2015 Finland’s disability pension (DP) expenditure was 2057 million euros, of which two-thirds were due to musculoskeletal (27% in 2015) and mental (41% in 2015) disorders [1]. Similarly, in the Nordic countries most long sickness absences are due to the same illness categories [1,2]. Supporting people to stay at work is perceived as important by governments [3,4]. Occupational health (OH) services play an important role in supporting individuals with lowered work ability in Finland [4]. Part-time solutions and changes in work descriptions are only part of the current solutions for supporting employees to remain in the workforce [5]. Sickness absences are known to predict DP [6,7] but other and earlier predictors of DP would be useful to steer individuals towards rehabilitation or new working careers before DPs are imminent.

Frequent attendance in healthcare is associated with the same illness categories in both general practice (GP) and OH primary care settings and with DP [8–10]. Frequent attenders in healthcare constitute a vulnerable group of patients that consume substantial healthcare resources. The organisational burden is well established – the top decile of attendees constitute up to 40% of physicians’ workload in primary care settings [10–12]. Frequent attendance is associated with chronic illnesses, unemployment and retirement [12,13] and often some combination of somatic, psychological and social problems [9,12,13]. Frequent attenders are sometimes subcategorised to differentiate between occasional-1-year-FAs (1yFA) and persistent frequent attenders (pFAs), as pFAs can have more complex problems and consume proportionally more resources [14]. Frequent attenders also have more and longer sickness absences than average primary care users [15,16]. Associations with future disability are however as yet unestablished although their characteristics indicate elevated risk of future DP.

In Finland, visiting OH primary care is associated with illnesses related to diminishing work ability [17]. In addition, employees with long-term illnesses and contact with a physician for work-related issues are at an increased risk of future sick-leave of over one month in duration[18]. These findings suggest that frequent attenders in OH primary care could be a risk group for work disability. Although frequent attendance in GP settings has been established as being associated with being on (disability) pension [19], research is sparse on how frequent attendance is linked to future disability in the working population. A Swedish study in a GP setting showed increased risk of long-term sick-leave in 1yFAs ^16^ compared with non-FAs. On the other hand, a Scottish study demonstrated an increased consultation frequency three years prior to a disability allowance claim [20]. Despite these findings, it remains unclear whether the causes of frequent attenders’ early retirement are similar to other DP recipients, and whether 1yFAs and pFAs differ in this aspect. High attendance rates could also be used to detect those individuals that need rehabilitative interventions to prevent disability, even before long absences occur. Understanding the association between frequent attendance and future disability would allow for purposefully designed and timely activities and follow-up plans for working age patients in both GP and OH primary care settings.

The aim of this study is to determine whether frequent attendance is associated with risk of future disability grants and whether 1yFAs and pFAs differ in their risk of DP.

## Material and methods

### Study setting and design

In Finland, OH is an important primary care provider for the working population, functioning side by side with municipal and private primary care services. Approximately 90% is entitled to OH primary care, with most costs covered by the employer [21]. Most staff in OH primary care have OH specialisation, supporting the preventive functions of OH services [22]. An example of such work is OH collaborative negotiation, a confidential negotiation between the patient, employer and OH physician to discuss work ability and possible solutions [23].

DP may be granted in Finland for individuals whose work ability has been reduced due to an illness for at least a period of one year. Partial fixed-term and fixed-term DPs are granted when rehabilitation is expected and for the duration of the rehabilitation. For a full DP (fixed-term or permanent) work ability must be reduced by at least 3/5 and for partial disability benefit (fixed-term or permanent) by 2/5 based on a physician’s assessment [1]. In addition, vocational rehabilitation allowance may be used to change occupations, when an employee cannot continue in their previous work. Permanent full DP leads to withdrawal from the workforce. DPs are funded by a mandatory insurance paid by employees and employers.

This is a longitudinal retrospective study combining routine medical record data with register data. This study was conducted using Pihlajalinna Työterveys’ data from the years 2014−2016. Pihlajalinna operates nationwide in rural and urban areas providing OH services for private and municipal employers. The clientele is fairly representative of the working population in Finland. Several corporate acquisitions were conducted during the study years, which increased the study population. We obtained the decisions on DP benefits (2015–2017) from the Finnish Centre for Pensions (FCP).

### Data collection

Pihlajalinna’s data were collected and pseudonymised by Pihlajalinna and sent to Tampere University. Medical record data included visits to physicians, nurses, physiotherapists and psychologists, the mandatory first diagnostic code (ICD-10) recorded for each physician visit, sickness absence certificates given on a visit, OH negotiations held and background data including patient age and sex, and employer size and industry. Data obtained from the FCP included decisions on disability benefits and the diagnostic codes associated with the decision [1]. The data from the FCP were combined using a pseudonymised ID-number, and the pseudonymised data were sent to Tampere University.

**Figure 1.**
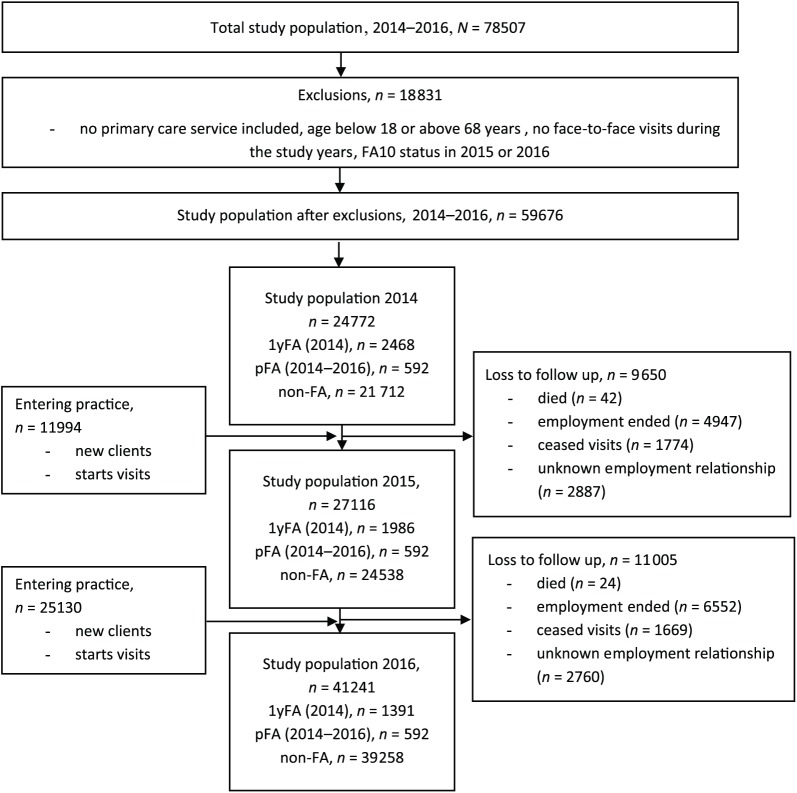
Flow chart of the study population. 1yFA = Patients being in the top decile of attenders in 2014. pFA = Patients being in the top decile in all three study years (2014, 2015 and 2016). non-FA = Patients that were never in the top decile were considered as a reference group, non-frequent attenders.FA10 = FA status defined as the top decile of attenders (frequent attender 10%, FA10).

Our data initially comprised 78,507 patients. We limited the study population to employees aged 18–68 years with at least one face-to-face visit to the OH unit. Any general and mandatory health check-ups and contacts not conducted face-to-face (prescription renewals, telephone calls, etc.) were excluded. After exclusions the study population comprised 59,676 patients (Figure 1). There were no missing data.

### Statistical analysis

Frequent attenders were defined as the top decile of attendees per year [11,24]. This meant eight or more visits in a year [10]. The remaining 90% were categorised as non-frequent attenders (non-FAs). Visits to physicians, nurses, physiotherapists and psychologists were used to define frequent attenders. Patients being frequent attenders in 2014 but not after this were categorised as 1yFAs. Patients being frequent attenders during 2014−2016 were categorised as pFAs . Patients that were never frequent attenders were used as a reference group (non-FAs). To account for confounding, patients being frequent attenders in 2015 or 2016 but not during all study years were excluded as they neither represented 1yFAs nor pFAs, nor could they be considered non-FAs.

The study population was divided by sex and into four age categories. Employer industries were categorised according to Statistics Finland (TOL2008/Nace Rev2). We used chi square to test for significant differences between the studied groups. Kaplan–Meier survival curves with stratification of FA status and the log-rank test were used to analyse durations of sickness absence before DP for the different FA groups. We used the total number of sickness absence days (2014–2016) as the follow-up time.

The main outcome was permanent DP as registered on FCP registry. Secondary outcome measures included partial fixed-term DP, partial DP, fixed-term DP and vocational rehabilitation allowance. Odds ratios (ORs) with 95% confidence interval (CI) were calculated for all outcome measures for the FA groups. The results were adjusted for patient age and sex, employer industry, number of different ICD-10 diagnoses, a cancer dummy variable and number of preceding sickness absence days. Statistical analyses were conducted at Tampere University using *R* and IBM’s SPSS. Alpha was set at 0.05.

### Ethical considerations

The study was approved by the Pirkanmaa Hospital District Ethics Committee (ETL R16041) and the National Institute of Health and Welfare (THL/556/5.05.OO/2016). Based on Finnish legislation, individual consent is unnecessary since no individual could be identified due to the size of the study population.

## Results

The study population comprised 59,676 patients during 2014−2016. There were 592 pFAs and 2468 1yFAs in 2014 (Figure 1). Due to loss to follow-up, the latter group of 1yFAs diminished so that in 2015 there were 1986 individuals and in 2016 1391 individuals in the 1yFA group. Men constituted 46%, 44% and 57% of patients for 1yFA, pFA and non-FA respectively (Table I).

**Table I.** Characteristics by frequent attender status: 1yFAs, pFAs and non-FAs yearly (2014–2016).

	Patients 2014−2016, *n* = 59,676						*p*-value
	1yFA *n* = 2468		pFA *n* = 592		non-FA *n* = 56,616		
	*n*	%	*n*	%	*n*	%	
Sex							<0.001
Male	1134	46	262	44	32,566	57	
Female	1334	54	330	56	24,050	43	
Age							<0.001
18–34	631	26	108	18	18,494	33	
35–44	546	22	132	22	13,218	23	
45–54	628	25	188	32	13,996	25	
55–68	663	27	164	28	10,908	19	
Disability grants (2015−2017)							<0.001
Permanent disability pension	67	2.7	13	2.2	214	0.4	
Partial disability pension	34	1.4	24	4.1	140	0.2	
Fixed-term disability pension	37	1.5	13	2.2	197	0.3	
Partial fixed-term disability pension	8	0.3	6	1.0	49	0.1	
Vocational rehabilitation	91	3.7	32	5.4	298	0.5	
OH collaborative negotiation	382	15.5	163	27.5	588	1.0	

OH = occupational health.

FA status was defined as the top decile of attenders (frequent attender 10%, FA10); 1yFA = patients being in the top decile of attenders in 2014; pFA = patients being in the top decile in all three study years (2014, 2015 and 2016); non-FA = patients that were never in the top decile were considered as a reference group, non-frequent attenders.

Proportionally 1yFAs received the greatest number of permanent DP decisions and non-FAs the least (2.7% of 1yFAs, 2.2% of pFAs and 0.4% of non-FAs) as seen in Table I. The pFA group received, proportionally, the most vocational rehabilitation allowances and partial or fixed-term disability resolutions. During the follow-up period 14.9% of pFAs, 9.6% of 1yFAs and 1.6% of non-FAs had any disability pension decision (*p* < 0.001).

Almost half of permanent DP decisions awarded to pFAs and 1yFAs were given based on musculoskeletal diseases (55% and 46% respectively) and for 31% of non-FAs (Table II). For pFAs, 23% of decisions were made based on mental disorders (16% for 1yFAs and 12% for non-FAs). In the group of non-FAs the second largest group was C00-D48 neoplasms (17%). The proportion of neoplasms leading to permanent DP was 8% for pFAs and 9% for 1yFAs. For any DP decision, diseases of the musculoskeletal system constituted 59% of decisions for 1yFAs and pFAs and 39% for non-FAs. The second largest group leading to any DP was mental and behavioural disorders with a 16%, 14% and 21% share for 1yFAs, pFAs and non-FAs respectively.

**Table II.** Distribution of diagnostic codes leading to disability pension decisions (2015–2017), *n* = 1223.

	Any DP by FA status						*p*-value	Permanent DP by FA status						*p*-value
	1yFA *n* = 237		pFA *n* = 88		non-FA *n* = 898			1yFA *n* = 67		pFA *n* = 13		non-FA *n* = 214		
ICD-10	*n*	%	*n*	%	*n*	%		*n*	%	*n*	%	*n*	%	
C00–D48 Neoplasms	13	5	3	3	79	9	***	6	9	1	8	36	17	***
F00–F99 Mental and behavioural disorders	37	16	12	14	185	21	***	11	16	3	23	26	12	***
G00–G99 Diseases of the nervous system	18	8	4	5	73	8	***	5	8	0	0	26	12	***
I00–I99 Diseases of the circulatory system	4	2	8	9	76	8	***	1	2	1	8	30	14	***
M00–M99 Diseases of the musculoskeletal system and connective tissue	141	59	52	59	350	39	***	37	55	6	46	66	31	***
Others	23	10	9	10	135	15	***	7	10	2	15	30	14	***
All	237	100	88	100	898	100	***	67	100	13	100	214	100	***

*** = < 0.001.

ICD-10 = International Classification of Diseases 10^th^ edition.

DP = disability pension.

FA status was defined as the top decile of attenders (frequent attender 10%, FA10); 1yFA = patients being in the top decile of attenders in 2014; pFA = patients being in the top decile in all three study years (2014, 2015 and 2016); non-FA = patients that were never in the top decile were considered as a reference group, non-frequent attenders.

Table III shows the OR for different DPs. Crude ratios indicate that pFAs and 1yFAs have increased risk of any disability grant when compared with non-FAs. These associations appear to be accentuated when adjusting for sex, age, field of industry, number of different ICD-10 diagnoses and the cancer dummy. When the ratios are also adjusted for the total number of preceding sickness absence days, the group of 1yFAs have an increased risk of partial DP (OR 2.26, 95% CI 1.36–3.76) and vocational rehabilitation allowance (OR 1.89, 95% CI 1.29–2.78) compared with non-FAs. In the adjusted analyses the pFA group also has increased risk of partial DP (OR 6.02, 95% CI 3.02–12.00) compared with non-FAs, while the risk of permanent DP is smaller (OR 0.12, 95% CI 0.05–0.29). When comparing groups of pFAs and 1yFAs, pFAs have a lower risk of permanent DP (0.21, 95% CI 0.10–0.45).

**Table III.** Different pensions associated with frequent attendance in multinomial logistic regression, *n* = 59,676.

	Crude ratios						Adjusted ratios: Model 1						Adjusted ratios: Model 2					
	1yFA vs. non-FA		pFA vs. non-FA		pFA vs. 1yFA		1yFA vs. non-FA		pFA vs. non-FA		pFA vs. 1yFA		1yFA vs. non-FA		pFA vs. non-FA		pFA vs. 1yFA	
	OR	95% CI	OR	95% CI	OR	95% CI	OR	95% CI	OR	95% CI	OR	95% CI	OR	95% CI	OR	95% CI	OR	95% CI
Partial fixed-term disability pension	3.75	1.78 - 7.94	11.82	5.04 - 27.70	3.15	1.09 - 9.11	4.68	2.03 - 10.79	28.73	9.06 -91.11	6.14	1.86 - 20.28	0.71	0.28 - 1.84	2.38	0.64 - 8.86	3.34	0.97 - 11.55
Fixed-term disability pension	4.36	3.06 - 6.21	6.43	3.65 - 11.34	1.48	0.78 - 2.79	5.11	3.36 - 7.75	10.59	5.18 -21.64	2.07	1.03 – 4.16	0.73	0.42 - 1.28	0.57	0.24 - 1.39	0.78	0.37 - 1.68
Partial disability pension	5.64	3.87 - 8.22	17.05	10.97 - 26.49	3.03	1.78 - 5.14	4.45	2.85 - 6.94	15.44	8.44 - 28.26	3.47	1.93 - 6.24	2.26	1.36 - 3.76	6.02	3.02 - 12.00	2.66	1.46 - 4.87
Permanent disability pension	7.36	5.57 - 9.71	5.92	3.36 - 10.42	0.81	0.44 - 1.47	7.83	5.54 - 11.06	7.64	3.84 -15.21	0.98	0.51 - 1.89	0.56	0.34 - 0.92	0.12	0.05 - 0.29	0.21	0.10 - 0.45
Vocational rehabilitation allowance	7.24	5.70 - 9.18	10.80	7.43 - 15.70	1.49	0.99 - 2.26	9.31	6.96 - 12.45	17.76	10.76 - 29.33	1.91	1.19 – 3.05	1.89	1.29 - 2.78	1.63	0.89 - 2.96	0.86	0.52 - 1.43

Model 1 adjusted for sex, age, field of industry, number of different ICD 10-diagnoses and cancer dummy.

Model 2 adjusted for the same as above (sex, age, field of industry, number of different ICD 10 -diagnoses and cancer dummy) and total number of sickness absence days.

OR = odds ratio; CI = confidence interval.

FA status was defined as the top decile of attenders (frequent attender 10%, FA10); 1yFA = patients being in the top decile of attenders in 2014; pFA = patients being in the top decile in all three study years (2014, 2015 and 2016); non-FA = patients that were never in the top decile were considered as a reference group, non-frequent attenders.

**Figure 2.**
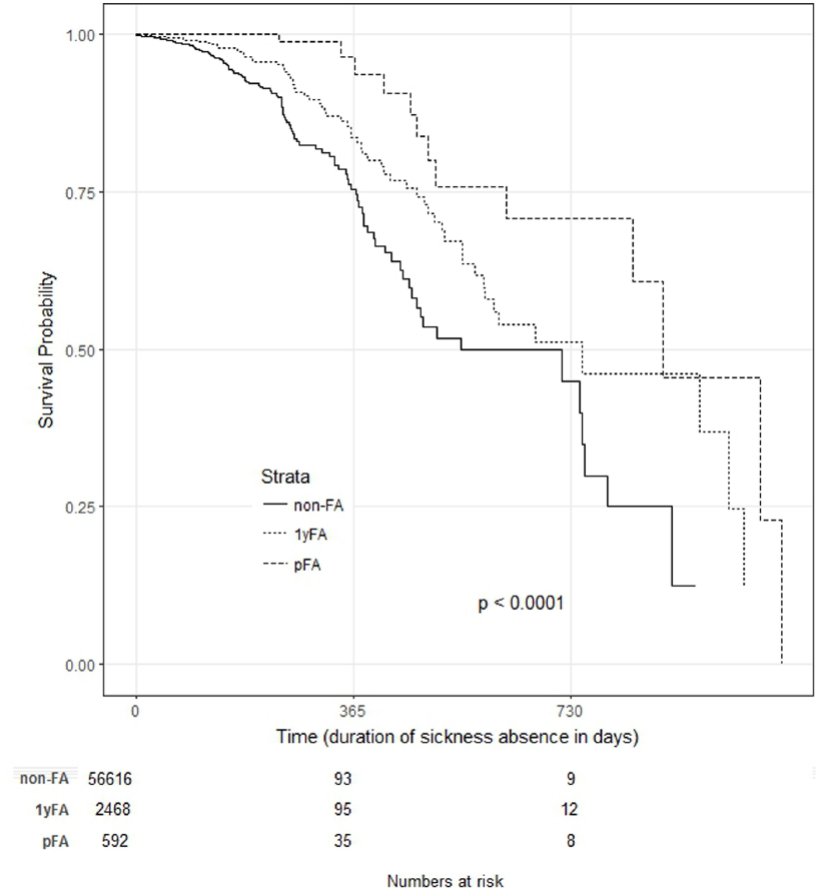
Kaplan–Meier survival curves with stratification of clients’ status (1yFA, pFA and non-FA) starting from the first sickness absence day of each individual (only patients with a sickness absence) and ending in permanent DP. Each drop on the curve indicates an individual receiving a DP. Half of each group (50%) had received a DP decision at 546 days (non-FA), 750 days (1yFA) and 886 days (pFA). 1yFA = Patients being in the top decile of attenders in 2014 pFA = Patients being in the top decile in all three study years (2014, 2015 and 2016) non-FA = Patients that were never in the top decile were considered as a reference group, non-frequent attendersDP = disability pension

Although there are more DP grants for 1yFAs and pFAs as a whole, the time delay before the DP grant is significantly longer for pFAs and 1yFAs compared with non-FAs (Figure 2). Each drop on the curve indicates an individual receiving a DP. Half had received a DP at 546 days (non-FAs), 750 days (1yFAs) and 886 days (pFAs). The group of pFAs had significantly more sickness absence days (median 490) prior to disability grant than the other two groups (1yFAs median 309 and non-FAs median 61 days, *p* < 0.001).

## Discussion

Our results show that frequent attenders, both 1yFAs and pFAs, have proportionally more DPs in the near future than average user of OH primary care. Most permanent DP grants leading to withdrawal from the workforce are granted for 1yFAs, followed by pFAs. On the other hand, permanent pFAs have proportionally more partial and fixed-term DPs and vocational rehabilitation decisions than 1yFAs and non-FAs, allowing for return to the workforce. However, the elevated risk of DP of both the frequent attender groups is mostly due to the preceding sickness absence days.

To our knowledge this study is the first to examine the differences between 1yFAs and pFAs and the distribution of diagnoses leading to DP among these groups. Our results show that high consultation frequency in the OHS, even occasional, is associated with DP in the following years. Proportionally, 1yFAs received the most permanent DP decisions and non-FAs the least. The increased risk of DP among the FA groups is for the most part explained by elevated sickness absence days, which has been shown to be a strong indicator of DP risk [6,7]. In previous work, frequent attendance was associated with long sickness absences in GP [16] and OH settings [15]; frequency of consultation could therefore potentially be used as an early marker for rehabilitative needs before sickness absences develop.

This study also showed that pFAs have more vocational rehabilitation resolutions and partial and fixed-term DPs than other users, indicating that temporary resolutions are sought for them more frequently than for non-FAs and 1yFAs. Thus, although there are more DPs given as a whole, pFAs and 1yFAs may take more advantage of possibilities that allow for remaining in and returning to the workforce. DPs shorten working careers in Finland by approximately 11 years [25]. Fixed-term DPs are used increasingly to enable a return to employment [26] and only approximately half of these lead to permanent disability in 4 years [26]. As an alternative for permanent resolutions, fixed-term resolutions facilitate a return to work after recovery. There are several possible explanations for the distribution of DP types between the frequent attender groups, including diagnosis-related reasons and the positive effects of OH measures, however further research is needed to establish the reasons. Almost one-third of pFAs had attended OH collaborative negotiation, while only 16% of 1yFAs and 1% non-FAs had done so. As OH collaborative negotiation is the place to discuss work modifications [23], it is possible that workplace interventions and other measures prior to disability application are used more often for clients who attend them. This might also postpone applying for DPs, possibly explaining pFAs’ longer sickness absences before DP.

The distribution of diagnoses leading to permanent DP in our study differs slightly from the general distribution reported by the FCP [27]. Over half of the DPs awarded for 1yFAs and pFAs are based on musculoskeletal disorders, while in 2017 FCP statistics covering all decisions in Finland, the proportion was less than a third [27]. This is similar to the proportion of non-FAs. This suggests that 1yFAs and pFAs leave the workforce due to musculoskeletal disorders more often than the average user of OH services. On the other hand, only 16% of 1yFAs and 12% of non-FAs retired due to mental disorders, while FCP statistics show that on average 30% of permanent DPs are awarded based on mental disorders [27]. In this study, mental disorders led to permanent withdrawal from the workforce less than in the FCP statistics, which might be due to the study population solely consisting of the working population, excluding the unemployed. It is also possible that the patients suffering from the more severe mental disorders mental disorders that finally lead to DP attended other services besides OH. Further research is needed on the use of other healthcare sectors to grasp the entire picture of disability caused by these illnesses that can be managed in multiple service sectors. Neoplasms leading to DP usually cannot be solved by the OHS nor partial DP solutions and are more common with the non-FA group as their care is usually coordinated in secondary care.

Measures that help to lengthen working careers and postpone DPs are welcome in the current economic situation and age-structure of Western and Asian countries such as Japan. Including frequency of consultation in the selection criteria of rehabilitation programmes could allow for earlier interventions for those at risk of DP, rather than relying solely on sickness absence rates. Authors have previously argued that 1-year frequent attenders should be excluded from interventions aimed at frequent attenders, as their frequency of visits diminishes on its own [28]. However, our results indicate that 1yFAs have proportionally more permanent DPs than permanent pFAs do, which indicates a decline in work ability. To date, interventions aimed at frequent attendance have focused mainly on morbidity and reduction of consultations rates [29]. Our results indicate, however, that frequent attenders’ work ability, and interventions aimed at improvement of working ability should also be considered. Careful evaluation of rehabilitative needs and multi-professional interventions, including care coordination, should be made. Frequency of consultation should be considered as an early indicator of DP risk when choosing groups for OH interventions aimed at reducing sickness absences or future disability, especially in subgroups of musculoskeletal and mental disorders.

Our study also has some limitations. We could not control for income, occupational status or level of education as they are not available through medical records. We did not have access to data on the use of other healthcare services such as the public sector or secondary care, or different OH providers. However, previous research indicates that when OH primary care services are available they are often used as the sole primary care provider [30]. In OH services loss to follow-up is possibly larger than in GP settings due to the ending of occupational relationships. Furthermore, we could not track the service use of patients lost to follow-up. This might have increased inaccuracy of the categorisation of different frequent attender groups. In a previous study, we conducted confirmatory analyses on the subgroup of 1391 1yFAs whose service use was known for the entire study period. The results did not differ substantially. The strengths of this study include the longitudinal study design that allowed for examining risks associated with both occasional and persistent frequent attendance. Moreover, the large study population from a nationwide OH service provider covers a wide range of industries and company sizes allowing for careful generalisation outside this particular context. The distribution of company sizes and industries resembles that of Statistics Finland. The health registers in Finland are comprehensive and accurate allowing for quality data.

## Conclusions

Frequent attenders of OH primary care receive proportionally more DPs than other users of OH primary care. Their increased risk of DP is explained by their sickness absences. High consultation frequency appears to indicate potential disability risk and careful rehabilitative assessment and care-planning should be conducted. Frequency of consultation could be considered when choosing candidates for early rehabilitation aimed at reducing DPs, especially in musculoskeletal and mental disorders, where the supportive measures of employers and OH services can be used. Further research is needed on working age frequent attenders using all parallel service providers. A longer follow-up period to evaluate risk of DP in the long term would be useful. Rehabilitative interventions aimed at working age frequent attenders of the OH services should be examined keeping in mind disability evaluation.
